# Evaluating Repetitive Transcranial Magnetic Stimulation for Refractory Chronic Cluster Headache Prevention: Insights from a Randomized Crossover Pilot Trial

**DOI:** 10.3390/brainsci15060554

**Published:** 2025-05-23

**Authors:** Leonardo Portocarrero-Sánchez, Cristian Rizea, Exuperio Díez-Tejedor, Moisés León-Ruiz, Javier Díaz-de-Terán

**Affiliations:** 1 Neurology Department, University Hospital La Paz, 28046 Madrid, Spain; leonardo9493@gmail.com (L.P.-S.); cristian.rizea@salud.madrid.org (C.R.); exuperio.diez@salud.madrid.org (E.D.-T.); pistolpete271285@hotmail.com (M.L.-R.); 2 Hospital La Paz Institute for Health Research, IdiPAZ, La Paz University Hospital, Universidad Autónoma de Madrid, 28046 Madrid, Spain

**Keywords:** cluster headache, chronic, refractory, neuromodulation, repetitive transcranial magnetic stimulation

## Abstract

**Background/Objectives**: Cluster headache (CH) is a debilitating primary headache disorder characterized by severe unilateral pain attacks. Chronic CH (CCH) poses significant treatment challenges, especially in refractory cases. Neuromodulation, including repetitive transcranial magnetic stimulation (rTMS), offers a potential alternative; however, evidence for its efficacy in CCH is lacking. **Methods**: A randomized, double-blind, placebo-controlled, crossover pilot study was conducted. Eligibility criteria included patients with refractory CCH (rCCH), who were then randomized to receive two treatment sequences: A, rTMS followed by sham stimulation, or B, sham followed by rTMS, separated by a one-month washout, with a follow-up period of 3 months. The primary endpoint was to analyze efficacy by assessing the change in the number of attacks per week (APW). Secondary endpoints included treatment tolerability and changes in intensity, duration, and use of rescue medication. The trial was registered with ClinicalTrials.gov (NCT06917144). **Results**: Eight patients were enrolled and randomized with a 50% probability of assignment to either treatment arm. Despite this, five patients were allocated to sequence A and three to sequence B. Three patients completed the entire study; five received treatment with rTMS and six with sham. The APW change during rTMS showed a change of (mean ± SD) +2.2 (10.8) attacks per week (*p* = 0.672). Two patients achieved complete remission during the rTMS phase, though symptoms returned by the washout period. In comparison with sham, the difference was also not statistically significant. No significant changes were observed in secondary endpoints. Side effects (two cases) were mild and transient. **Conclusions**: This pilot study suggests that rTMS may provide clinical benefits for rCCH in selected cases, though its effects seem transient. Adherence to treatment remains a critical challenge.

## 1. Introduction

Cluster headache (CH) is a primary headache disorder characterized by severe, strictly unilateral pain localized to the orbital, supraorbital, and/or temporal regions. These attacks typically last between 15 and 180 min and occur in clusters or “bouts.” Based on the duration of pain-free intervals, CH is classified as either episodic (ECH), where bouts are separated by pain-free periods lasting more than three months, or chronic (CCH), where pain-free intervals last less than three months or are absent altogether [1].

Pharmacological treatment is the cornerstone of CH management. However, a subset of patients fail to achieve adequate symptom control despite therapy [2], leading to refractory chronic CH (rCCH), as defined by the European Headache Federation (EHF) in 2014 [3]. These refractory cases are associated with significant disability and limited treatment options, necessitating alternative approaches such as neuromodulation [4].

Neuromodulation involves the targeted modulation of the nociceptive system and can be performed invasively (e.g., occipital nerve stimulation (ONS) or deep brain stimulation (DBS)) or non-invasively (e.g., vagus nerve stimulation or transcranial magnetic stimulation (TMS)) [5]. Among these, TMS is particularly promising due to its non-invasive nature and ability to modulate cortical activity [6,7,8].

TMS operates on the principle that an electric current generates a perpendicular magnetic field, which penetrates the skull without significant attenuation and induces cortical depolarization [6]. Depending on the type of coil used, the stimulation can be highly localized. Analgesia is achieved through mechanisms that include the activation of distant neuronal structures.

Although the optimal stimulation site for CH remains unclear, studies on other headache disorders, such as migraine, suggest that targeting the primary motor cortex (M1) or dorsolateral prefrontal cortex (DLPFC) may provide analgesic effects [9,10,11,12]. Overall, in chronic migraine, there were more positive results from performing rTMS on M1 than DLPFC [10,13]. TMS can be delivered as a single pulse or as repetitive trains of pulses (repetitive TMS (rTMS)). Repetitive TMS, in particular, has demonstrated sustained effects at the cellular and biochemical levels and is an established therapy for several neurological conditions, including migraine [6,7,8,14]. Indeed, a promising emerging therapeutic option for drug-refractory chronic migraine with favorable results is neuromodulation, including rTMS [15].

In a recent systematic review and meta-analysis of pharmacotherapy and non-invasive neuromodulation for neuropathic pain, among the neuromodulation studies that met the inclusion criteria, rTMS was the most studied treatment, followed by transcranial direct current stimulation (tDCS). More specifically, 15 studies evaluated rTMS at several targets, predominantly M1 (12 studies). For rTMS, at M1, the combined number needed to treat (NNT) (six comparisons) was 4.2 (95% CI 2.3–28.3); the estimate of effect ( 14 comparisons) expressed as the standardized mean difference (SMD) was 0.9 (0.4–1.4); and the number needed to harm (NNH), based on those who received the intervention (12 comparisons), was 651.6 (34.7–∞). Removal of an outlier increased the NNT by 36% to 6.6 (3.67–31.97) and decreased the SMD by 15% to 0·8 (0.3–1.3). There was a low certainty of evidence [9].

However, the application of rTMS in CH, particularly rCCH, has been poorly studied.

Recently, it has emerged that high-frequency rTMS targeting M1 can reduce pain perception in patients with chronic pain [16]. However, the exact mechanisms underlying this pain reduction remain not completely elucidated. In healthy subjects, increased excitability of the corticospinal motor system induced by high-frequency rTMS can lead to more efficient inhibitory modulation of pain. Several hypotheses exist regarding the mechanisms through which rTMS reduces pain, including thalamic activation, which suppresses sensory information transmission via the spinothalamic pathway, as well as the activation of specific brain areas involved in descending pain modulation systems, such as the brainstem, anterior cingulate cortex, and DLPFC [16]. Moreover, reward mechanisms involving the putamen, medial prefrontal cortex, and nucleus accumbens may play a role in suppressing the negative emotional and behavioural aspects associated with neuropathic pain [16].

In chronic migraine, the mechanisms of pain relief through the application of high-frequency rTMS to the M1 could be due to top-down modulation of the descending pain control system as functional connectivity is revealed between the motor cortex, thalamus, insula, anterior cingulate cortex, and periaqueductal gray [17]. Post-TMS increased levels of β-endorphins and dopamine, and changes in the levels of other neurotransmitters and neuromodulators have been documented [17]. In CH (including CCH), possible mechanisms of rTMS-induced analgesia encompass decreased cortical excitability, release of beta endorphins, changes in glutamine/glutamate levels, and effects on the hypothalamus [18]. Of note, the hypothalamus has been found to play a key role in CH [19,20].

To date, only one naturalistic study has explored rTMS in CH [21]. This study, which included an induction and maintenance phase, involved 55 patients, 19 of whom met the criteria for CCH. The results demonstrated a significant reduction in the number of attacks per week (APW). However, the induction phase varied across participants, and the study lacked a control group, limiting the generalizability of its findings.

Having thoroughly reviewed the literature, we confirm that the study presented here is the first clinical trial to investigate rTMS in rCCH. This study is novel in its design as a controlled crossover trial with a sham group. The primary aim was to evaluate the efficacy of rTMS as a preventive therapy in patients with rCCH, as measured by the reduction in the number of APW. Secondary objectives included assessing the reduction in symptomatic medication use, treatment tolerability, and improvements in other headache parameters, such as intensity and duration.

## 2. Materials and Methods

### 2.1. Participants

We included patients who met the following criteria: 18 years of age or older; diagnosed with CCH based on the International Classification of Headache Disorders, Third Edition [1]; and diagnosed with rCCH based on the European Headache Federation criteria of 2014 [3].

The exclusion criteria were a history of epilepsy, concomitant diagnosis of any other headache (if the patient was unable to differentiate between them), carriers of any electronic device, or any additional contraindication for rTMS, such as pregnancy [6,7].

### 2.2. Study Design

We conducted a randomized, double-blind, placebo-controlled, crossover pilot study at a specialized tertiary center for CH treatment in Spain. The rTMS devices were provided by Ionclinics for a duration of three months. The study was conducted according to the recommendations of the Helsinki Declaration [22] and the Good Clinical Practice guidelines and was approved by the Ethics and Clinical Research Committee of La Paz University Hospital (PI-6581). The trial was registered with ClinicalTrials.gov (NCT06917144).

In our study, a crossover design was chosen instead of a parallel one due to the low prevalence of patients with rCCH and the condition’s temporal stability, which reduces variability in baseline symptoms across treatment periods. By allowing each patient to serve as their control, this design aimed to maximize statistical power and minimize inter-individual variability in pain perception. However, this design has certain limitations, such as potential carryover and period effects, which could influence the final interpretation of the results. To address this, the longest feasible washout period was planned (see Section 2.6), along with an analysis of potential carryover effects, randomization, and an assessment of possible period effects. Additionally, this design is susceptible to patient dropout, representing another significant limitation. Despite these factors, a crossover design was selected to optimize the detection of treatment effects.

Additionally, none of the patients had previously received rTMS, ensuring a lack of prior knowledge of its effects, which allowed the implementation of sham stimulation. Both the patients and data analysts were blinded to the type of treatment administered.

Patients were recruited sequentially and randomized using a simple computer-generated randomization protocol, with a 50% probability of assignment to either treatment arm for each patient. To ensure full double-blind administration, both patients and outcome assessors remained completely blinded to treatment modality. Only the investigator administering the treatment during each session was aware of the assigned intervention (with parameters fixed after the first session).

### 2.3. Protocol

**Figure 1:**
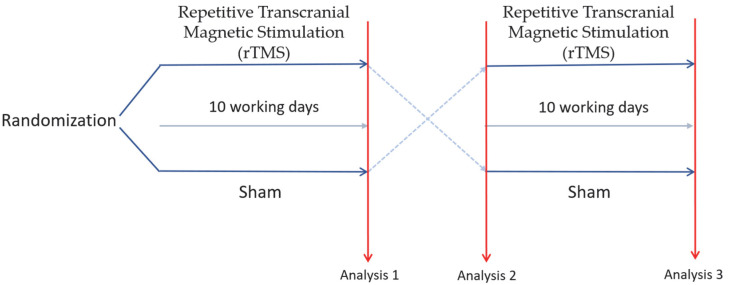
Protocol applied in the study.

The study consisted of two treatment periods (rTMS and sham stimulation) separated by a one-month washout period and organized into two sequences explained in Figure 1.

Sequence A:

rTMS → washout → sham.

Sequence B:

Sham → washout → rTMS.

Then, patients who completed both treatment periods were followed up for three months. The follow-up period was set at three months to observe, as extensively as possible, any potential treatment effects (should they occur) over a period longer than the established washout period.

### 2.4. Intervention Protocol

Stimulation sessions were performed using a YINGCHI M-100 Ultimate device (provided by Ionclinics, Valencia, Spain) with liquid-cooled figure-8 coils.

Patients attended 10 min sessions daily for 10 consecutive working days.

For rTMS, stimulation was delivered at an excitatory frequency of 10 Hz, targeting M1, contralateral to the side of pain in the facial region.

Each session consisted of the following steps.

- Ten series: 60 pulses per series (600 pulses in total).
- Rest intervals: 60 s between series.
- Intensity: 70% of the resting motor threshold (RMT).

The RMT was determined using single-pulse stimulation over the M1 region controlling the abductor pollicis brevis muscle, localized via neuronavigation. The threshold was defined as the lowest stimulator intensity producing a motor-evoked potential amplitude >50 µV in at least five of ten trials.

### 2.5. Sham Stimulation

The same device and protocol were used for sham stimulation, but no active stimulation was delivered. The equipment produced identical sounds and appearances, thereby ensuring blinding.

### 2.6. Washout Period

A one-month washout period was included between the treatment periods. Although no prior study has established the optimal duration, this timeframe was chosen based on the transient effects of rTMS observed in other studies, like the study conducted by Hodaj et al. [21], and logistical constraints.

In Hodaj et al.’s study [21], monthly maintenance sessions following the induction phase appeared to reduce the number of treatment responders. Other rTMS studies in chronic migraine reported effect durations of approximately 1.5 months (Shehata et al. [23]) and 3 months (Kumar et al. [13,17]). However, the number of sessions and administration frequencies varied across these studies.

### 2.7. Clinical Evaluation

Before the first session, all patients underwent a baseline clinical assessment. This assessment included demographic data, medical history, headache characteristics (such as attack frequency per week and pain intensity measured on a visual analog scale (VAS) from 1 to 10), and details of prior and current treatments.

Throughout the study (including the follow-up period), patients electronically recorded follow-up variables on their personal devices using a QR code linked to the REDCap platform for biomedical research. The recorded variables included attack frequency (per day), intensity, duration, use of rescue medication (triptans or oxygen, as per their usual treatment), adverse effects, and other secondary outcomes. If a patient experienced a pain attack, they were allowed to use their usual rescue medication. All collected data were reviewed before each treatment session.

### 2.8. Sample Size Determination

As this was a pilot study, no formal sample size calculations were performed.

### 2.9. Data Analysis

We analyzed the sociodemographic and clinical characteristics of the participants and summarized the data using frequency counts, descriptive statistics, summary tables, and figures.

Analyses were performed at different time points: at baseline; at the end of period 1; at the beginning of period 2; at the end of period 2; and at 2, 4, and 12 weeks after the end of the treatment sequence.

Data analysis was performed using the Statistical Package for Social Sciences (SPSS 25.00, IBM, Inc., Armonk, NY, USA). Categorical variables are presented as frequencies and percentages, while quantitative variables are expressed as descriptive statistics (means, standard deviations, and confidence intervals (CIs)). Given the small sample size (*N* < 30), the normality of the Z score distribution was assessed using the Kolmogorov–Smirnov and Shapiro–Wilk tests.

For discrete variables, comparisons between groups were performed using Pearson’s chi-squared test or Fisher’s exact test for small samples. For continuous variables, paired data were analyzed using the Student’s t-test when parametric assumptions were met; otherwise, the Wilcoxon test was applied. Additional tests were used as needed, depending on the characteristics of the variables studied.

## 3. Results

Patients were recruited sequentially during December 2023 and January 2024. Eight patients (six men, two women; mean (mean ± SD) age: 45.5 ± 3.67 years) were included. The male to female ratio was 3:1, in line with the most recent studies regarding the difference in the prevalence of CH depending on sex [24].

The baseline characteristics of the included patients can be seen in Table 1. No patient had structural brain injuries.

**Figure 2:**
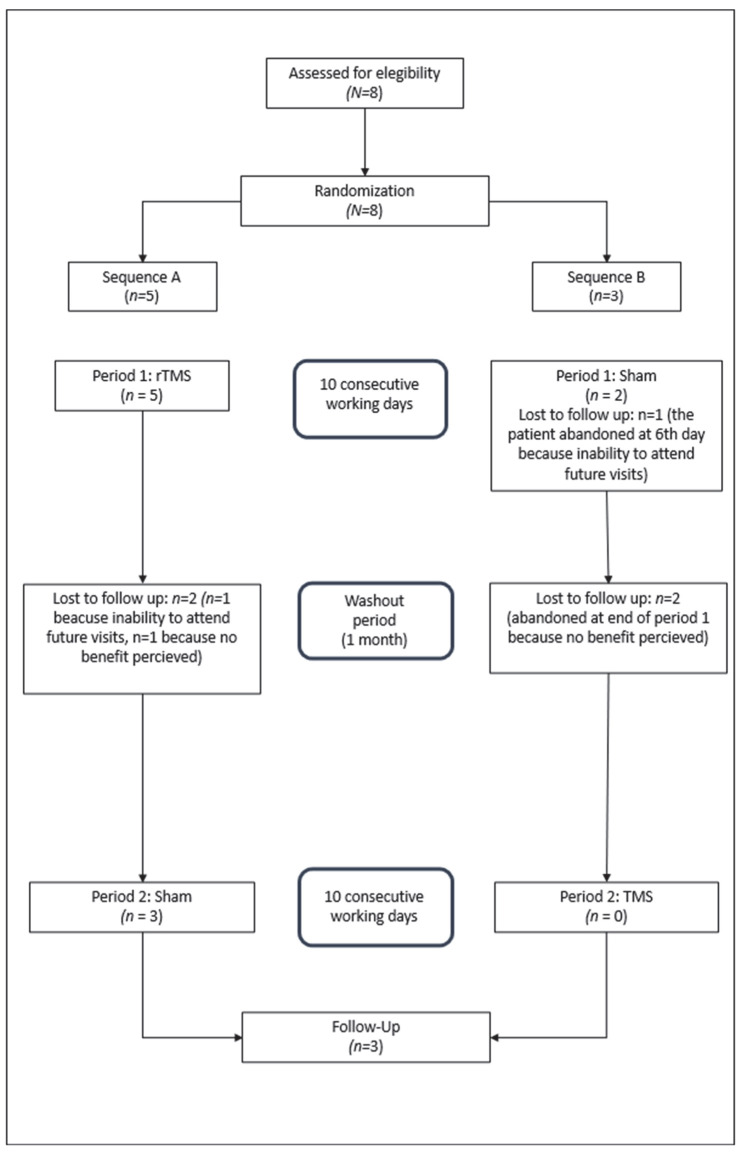
Flow diagram.

Figure 2 shows the study flow diagram according to the Consolidated Standards of Reporting of Trials guidelines [25,26].

Participants were sequentially randomized with a 50% probability for each treatment sequence using a simple computer-generated randomization protocol. Despite this, five were assigned to sequence A and three to sequence B.

Of the five patients randomized to treatment sequence A, five completed period 1, although two dropped out immediately after completion. The remaining three patients completed period 2 and the follow-up period.

Of the three patients allocated to sequence B, two completed period 1, with one patient discontinuing the study after the first 7 days. Data obtained up to that point were included in the analysis. The remaining two patients abandoned the study after completing period 1.

Among the five patients who dropped out, three did so due to poor willingness to comply with future visits and two cited a lack of response.

### 3.1. Efficacy Evaluation

When analyzing the change in the mean number of APW in the rTMS group (*n* = 5) using the paired t-test, there was an average increase of 2.2 (±10.8) attacks per week (*p* = 0.672).

Two patients showed a complete reduction in weekly attacks after day 4 of treatment; however, symptoms reverted to baseline by the seventh day of the washout period.

The change in the number of APW according to the treatment sequence can be observed in Table 2, and the characteristics of the two patients with complete remission are summarized in Table 3.

Comparison between treatment modalities (rTMS and sham) using the Wilcoxon test showed a Z score of −1.34, with no statistically significant difference (*p* = 0.180).

No residual effects were observed in patients in sequence A (*n* = 3) transitioning to period 2.

Since the patients in sequence B did not complete period 2, it was not possible to study the possible period effect or the possible interaction between the two treatments.

### 3.2. Secondary Outcomes

Regarding the study of secondary variables, no significant differences were found in medication consumption after treatment with rTMS (mean increase of nine uses (triptans/oxygen), with t = 0.71 and *p* = 0.5), duration (mean increase of 19 min, with t = 0.6 and *p* = 0.56), or intensity (reduction of two points in VAS, with t = −1.4 and *p* = 0.232). Table 4, “Outcome evaluations 2”, shows the detailed evolution.

### 3.3. Tolerability

Side effects were mild and transient, reported in two cases, without causing study withdrawal.

## 4. Discussion

This pilot study suggests that rTMS may offer clinical benefits to patients with rCCH, although statistical significance was not achieved. The therapeutic effect appeared to manifest relatively early during the rTMS treatment phase, with two patients achieving a complete response by day four, although this effect did not persist over time.

The study by Cosentino et al. [27] and the case reported by Kumar et al. [18] show effects of TMS in patients with CH, providing insights regarding both pathophysiology and its potential for therapy. Moreover, a systematic review and meta-analysis supports the potential of neuromodulation techniques, including rTMS, as effective preventive treatments for rCCH [4].

The previous literature on the use of rTMS in CH is limited to a single naturalistic study conducted by Hodaj et al. [21], which included 55 patients, 19 of whom met the CCH criteria. The study (induction phase of 12 sessions followed by a maintenance phase) reported a statistically significant reduction in attack frequency. Although our protocol resembled the induction phase of their study, we were unable to implement a longer maintenance phase owing to logistical constraints. This difference in design, coupled with the small sample size of our study (*n* = 5 completing sequence A), likely contributed to the lack of statistically significant findings.

A high dropout rate, primarily due to logistical challenges and lack of perceived benefit, limited the study’s sample size (three patients completing the full protocol). This reflects the difficulties in conducting research in rCCH patients, who often have high expectations of rapid relief. Compared to Hodaj et al.’s open-label study, our adherence rate was similar, though the inclusion of a placebo arm and masking may have influenced patient expectations.

Regarding the washout period, we observed no residual effects in this study. As previously mentioned in Section 2.6, no standardized washout duration has been clearly established. While prior studies suggest that rTMS effects may persist for 1–3 months, their administration protocols (number/frequency of sessions) differed from ours. On one side, maintenance of these effects in some patients indicate the establishment of neuroplasticity, which can be explained based on long-term potentiation (LTP) of pain-modulating areas that are functionally connected to the motor cortex [17]. On the other side, the transient treatment response observed in two patients, with a complete reduction in weekly attacks who then reverted to baseline by the seventh day of the washout period, probably reflect the need for a more sustained application of rTMS to consolidate the underlying biological and physiological rTMS mechanisms of action in this regard [6,7,8,9,10,11,12,13,14,15] which has been shown to trigger dose-dependent homeostatic rewiring in recurrent neuronal networks, highlighting the importance of network inhibition in careful protocol design, standardization, and optimization of stimulation [28].

Due to temporal constraints in rTMS machine availability, we set a 1-month washout period. However, with a larger sample size, a longer washout might be required to definitively exclude residual effects.

To our knowledge, this is the first controlled clinical trial of rTMS in rCCH. The potential benefit of rTMS in this pilot trial is supported by two patients achieving complete remission with treatment. Despite its limitations, the study highlights the potential of rTMS in select cases and emphasizes the need for further investigation through rigorously designed trials with appropriate methodologies to definitively establish rTMS efficacy in rCCH.

## 5. Clinical Implications

Patients with rCCH face significant morbidity, which profoundly affects their quality of life, largely due to the absence of effective preventive therapies. This study contributes to the limited body of literature on rTMS as a potential preventive treatment for rCCH. Although the primary endpoint was not achieved, the observed trend (especially in the two patients from sequence A) suggests that rTMS may warrant further exploration as a non-invasive therapeutic option. In line with the recommendations from the Spanish Society of Neurology and the Spanish Society of Neurosurgery, our study supports the use of neuromodulation techniques, such as rTMS, as a promising alternative for patients with rCCH who have exhausted pharmacological treatments [5]. Future studies should prioritize optimizing treatment protocols, including the incorporation of a maintenance phase, to enhance and sustain therapeutic effects.

## 6. Limitations

This study has several limitations which must be considered.

First, the small sample size reduced the statistical power of the study despite the crossover design aimed at mitigating this limitation which allowed each patient to serve as his or her own control. Thus, the findings should be interpreted cautiously. One possible way to improve this aspect for future studies would be to conduct multicenter collaborative research. An alternative approach would involve broadening the inclusion criteria to increase the sample size. As will be discussed later regarding the results’ generalizability, one option would be to include CCH patients who do not meet refractoriness criteria. Additionally, given the exploratory pilot nature of this study designed to assess the potential utility of rTMS in rCCH patients (a low-prevalence condition), a formal sample size calculation was not performed.

Second, treatment adherence was a major challenge, as there was a substantial dropout rate (63%). These two factors may have introduced biases (including effect estimation and selection biases) into the outcomes, requiring careful consideration when interpreting the results.

The effort required for daily attendance, combined with the severity of symptoms and lack of early-perceived benefits, likely contributed to this issue. To reduce dropout rates and enhance sample size/power, future studies might incorporate adherence-improving strategies, including a run-in phase design. Under such a methodology, all patients would first receive active rTMS treatment for a predefined period. Those demonstrating clinical benefit would then proceed to a double-blind phase, allowing placebo effects to be assessed. This approach would preclude non-responders from receiving sham stimulation, thereby optimizing trial efficiency.

Third, the absence of a maintenance phase limits our ability to evaluate the long-term effects of rTMS. Previous studies have highlighted the importance of maintenance sessions for sustaining therapeutic benefits. Future protocols should incorporate this phase, as clinical improvement achieved during acute treatment would be expected to persist with ongoing therapy. An open-label maintenance period would allow systematic exploration of this effect, followed by an observational washout phase to monitor disease evolution after treatment discontinuation. This design would enable a definitive assessment of rTMS’s true long-term impact. Therefore, large sham-controlled trials with extended, clinically relevant timeframes are strongly warranted.

Fourth, complementary measures, such as biomarkers (CGRP), imaging techniques (e.g., functional MRI), and motor evoked potentials (MEPs), that were not included in our study could be incorporated to enhance the robustness of findings and enable more comprehensive assessments.

Finally, this study exclusively included patients with rCCH to ensure a stable clinical presentation for the crossover design. Therefore, these findings cannot be generalized beyond this specific patient profile due to its unique characteristics. While potential applicability might be considered for ECH patients during a bout who have failed multiple preventive treatments, these patients differ in headache history, clinical features, and treatment responses. The closest comparable population would be CCH patients, though recent studies [2] report clinical differences between rCCH and CCH cases. Future studies could expand sample sizes by including CCH patients who do not yet meet rCCH criteria.

## 7. Conclusions

In conclusion, this pilot study indicated a potential clinical benefit of rTMS in the prevention of rCCH attacks, although its efficacy seems to be hard to maintain in the long term. Adherence to treatment is a key challenge.

## Figures and Tables

**Table 1:** Demographic clinical data.

	*N* = 8 Mean ± SD, Median, Frequency
Sex	75% men
Age	45.5 (3.67)
Age of onset (years)	31.38 (9.27)
Age of chronification (years)	35.75 (11.31)
Prior prophylactic * therapies	6.37 (4.4)
Occipital nerve stimulation (ONS)	25%
Current prophylactic * therapies	2.5 (1.69)
Number of attacks per week (APW)	18.13 (min. 3, max. 56), Median 12.5

* Prophylactic therapies for CH (for example, Verapamil, Topiramate, Lithium, etc.) according to current evidence-based guidelines at the time of the assessment.

**Table 2:** Outcome evaluations 1.

	Number of APW (Mean (SD); Median)						
	Baseline	End of Period 1	Baseline 2	End of Period 2	Follow-Up		
					W2	W4	W12
Sequence A, *n* = 5 (rTMS -> Sham)	19.8 (10.1)	22 (14.5)	5.67 (1.02) *	4.67 (1.2) *	6 (0.57) *	7.33 (0.88) *	6.67 (1.2) *
** *n* = 2	4.5 (2.12)	0	4.5 (0.7)	3.5 (0.7)	5.5 (0.7)	8 (1.4)	5.5 (0.7)
Sequence B, *n* = 3 (Sham -> rTMS)	15.3 (2.9)	17.3 (7.3) ***; 12	0 ****	0 ****	0 ****		

* Results obtained from *n* = 3 patients who completed both periods. ** *n* = 2. Patients with complete remission after period 1. *** Data include the results of a patient who dropped out after the first week. **** There are no results due to patients withdrawing from the study.

**Table 3:** Characteristics of patients who achieved complete remission.

	Age	Age of Onset	Age of Chronification	Prior Prophylactic Therapy	Current Prophylactic Therapy	Number of APW	Intensity (Visual Analog Scale (VAS))	Duration (min)	Use of Rescue Medication
Patient 1	61	45	54	3	6	6	8	20	12
Patient 2	43	28	34	7	2	3	3	15	0

**Table 4:** Outcome evaluations 2.

	(Mean (SD); Median)						
Sequence A, *n* = 5 (rTMS -> Sham)	Baseline	End of Period 1	Baseline 2	End of Period 2	Follow-Up		
					W2	W4	W12
Use of rescue medication per week	31.4 (14.87)	40.6 (25.9)	8.33 (4.63) *	6 (4.16) *	7,67 (4.09) *	8,33 (4.17) *	10 (5.29) *
** *n* = 2	6 (8.48)	0	4.5 (6.36)	2 (2.82)	4.5 (6.36)	6.5 (9.19)	6 (8.48)
VAS (1–10)	8	10	8 *	6 *	7 *	7 *	8 *
** *n* = 2	5.5 (3.5)	0	5.5 (3.5)	5.5 (0.7)	5 (2.8)	5 (2.8)	5.5 (3.5)
Duration (min)	37 (11.57)	56 (34.29)	16.67 (1.66) *	33.33 (13.33); 20 *	20 (0) *	16.67 (1.66) *	18.33 (1.66) *
** *n* = 2	17.5 (3.5)	0	17.5 (3.5)	40 (28.28)	20 (0)	17.5 (3.5)	17.5 (3.5)
Sequence B, *n* = 3 (Sham -> rTMS)							
Use of rescue medication per week	20.33 (5.84)	23.67 (9.28)	0 ****	0 ****	0 ****		
VAS (1–10)	10	10 ***	0 ****	0 ****	0 ****		
Duration (min)	35 (5);30	35 (5); 30	0 ****	0 ****	0 ****		

* Results obtained from *n* = 3 patients completing both periods. ** *n* = 2. Patients with complete remission after period 1. *** The results obtained from the patient who dropped out in the first week were included in the analysis. **** There are no results due to patients withdrawing from the study.

## Data Availability

The original contributions of this study are included in the article. Further inquiries can be directed to the corresponding authors.

## Abbreviations

The following abbreviations are used in this manuscript:

CH	Cluster headache
ECH	Episodic cluster headache
CCH	Chronic cluster headache
rCCH	Refractory cluster headache
APW	Attacks per week
rTMS	Repetitive transcranial magnetic stimulation
